# Supplementary figures and images for: Reticulon and CLIMP-63 regulate nanodomain organization of peripheral ER tubules
Source: PLoS Biol. 2019 Aug 30;17(8):e3000355. doi: 10.1371/journal.pbio.3000355 (PMC6742417; doi:10.1371/journal.pbio.3000355)

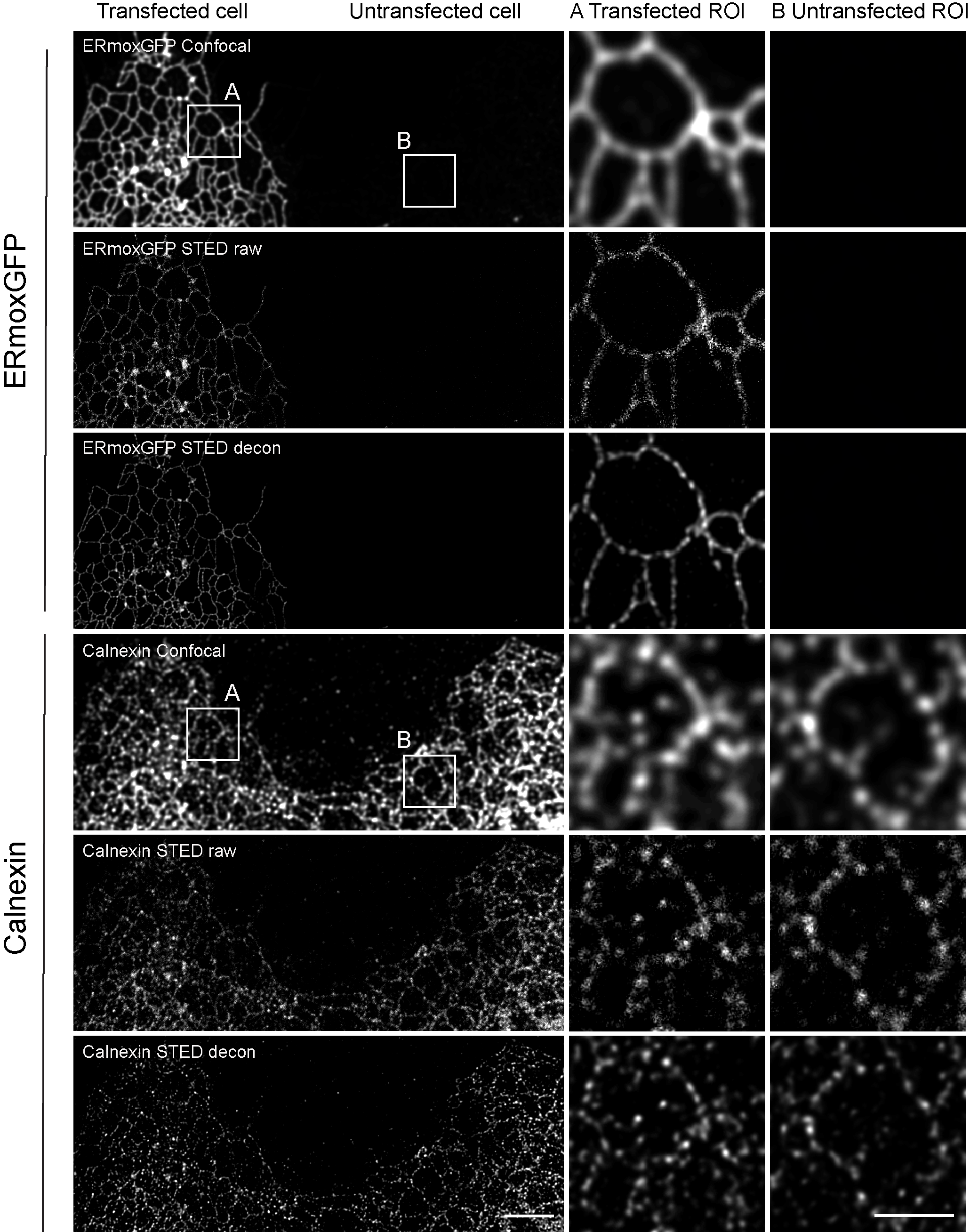

Supplement: S1 Fig — Representative confocal and STED images of calnexin and ERmomxGFP in ERmoxGFP transfected and untransfected cells. The punctate distribution of calnexin is observed more readily by STED (raw and decon) compared with confocal imaging in both (A) transfected and (B) untransfected cells. Scale bar, 5 μm; zooms, 2 μm. decon, deconvolution; ER, endoplasmic reticulum; ERmoxGFP, ER monomeric oxidizing environment-optimized green fluorescent protein; STED, stimulated emission depletion. (TIF) [file pbio.3000355.s001.tif]

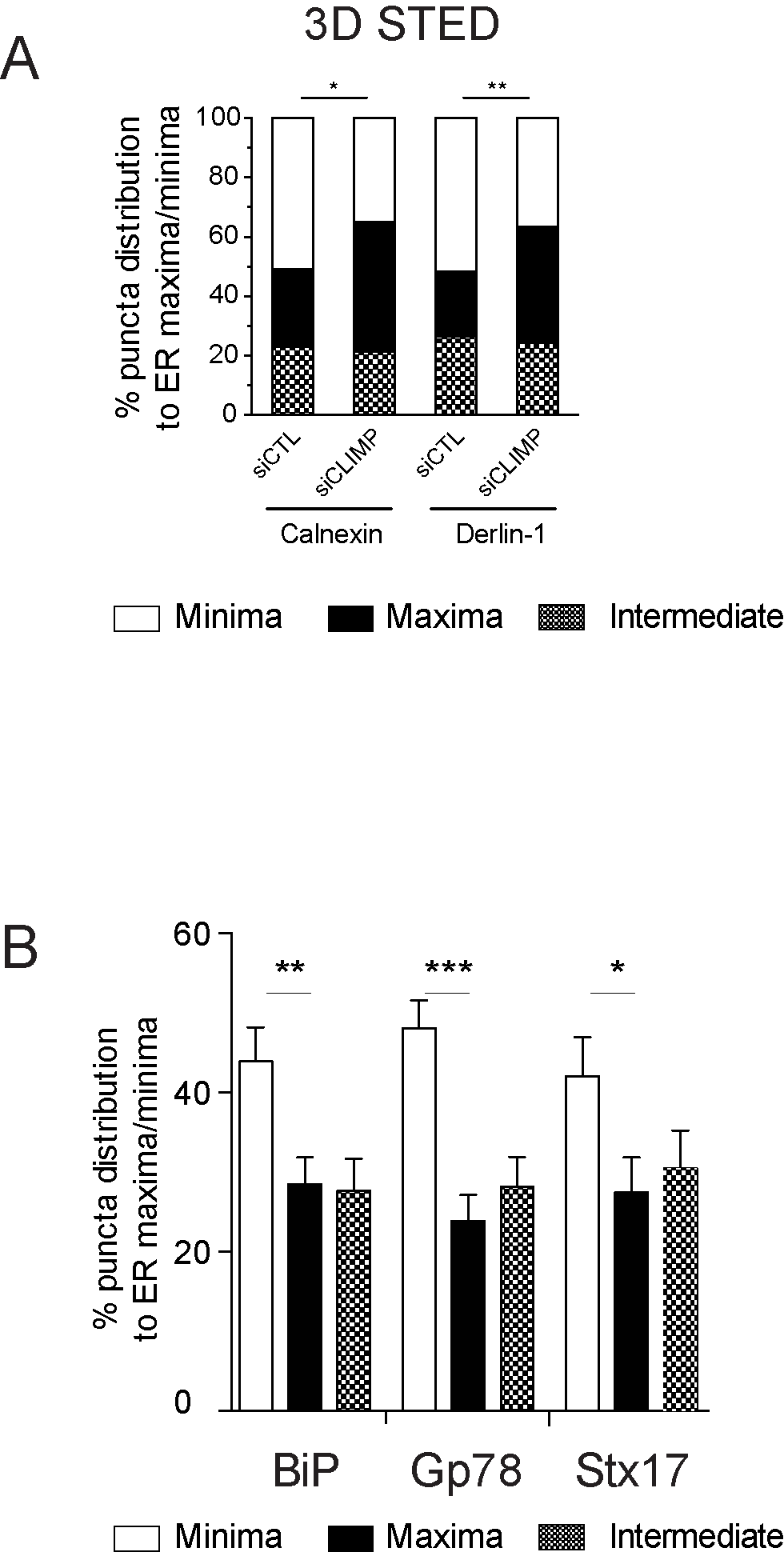

Supplement: S2 Fig — (A) Quantification of 3D localization of calnexin and derlin-1 puncta to ERmoxGFP maxima and minima of peripheral ER tubules in 3D STED images of siCTL or siCLIMP-63 HT-1080 cells. Significance assessed by χ2 test from at least 25 ROIs (2.5 um × 2.5 um) from 10 three-dimensional stacks for each condition at 2 degrees of freedom in three independent experiments. *P < 0.05; **P < 0.01. Numerical values that underlie the graphs are shown in S1 Data. (B) Based on line scan analysis of peripheral ER tubules imaged by 2D STED, localization of BiP, Gp78, and Stx17 puncta to ERmoxGFP maxima and minima was quantified. Significance assessed by one-way ANOVA from at least 20 line scans in three independent experiments. *P < 0.05; **P < 0.01; ***P < 0.001. Numerical values that underlie the graphs are shown in S1 Data. BiP, binding immunoglobulin protein; ER, endoplasmic reticulum; ERmoxGFP, ER monomeric oxidizing environment-optimized green fluorescent protein; Gp78, glycoprotein 78; ROI, region of interest; siCTL, siControl; siCLIMP-63, siRNA to CLIMP-63; STED, stimulated emission depletion; Stx17, Syntaxin-17; 2D, two-dimensional; 3D, three-dimensional. (TIF) [file pbio.3000355.s002.tif]

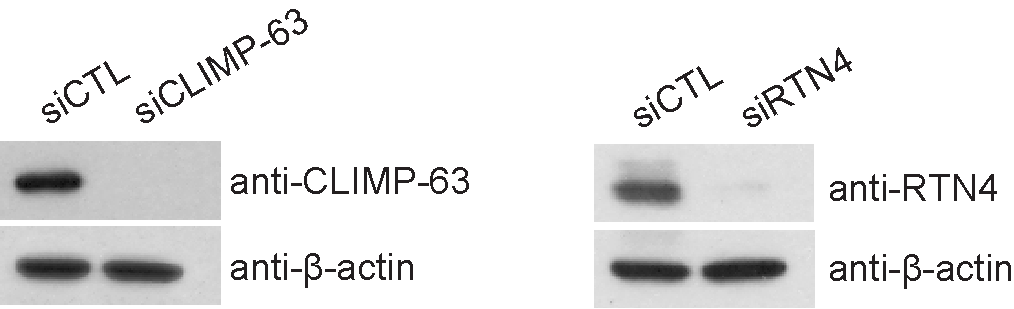

Supplement: S3 Fig — Western blots of RTN4 and CLIMP-63 siRNA knockdown in COS-7 cells. The blots were probed with anti-CLIMP-63, anti-RTN4, or anti-β-actin as a loading control. CLIMP-63, cytoskeleton-linking membrane protein 63; RTN4, reticulon4; siRNA, small interfering RNA. (TIF) [file pbio.3000355.s003.tif]
